# Characterization of Maillard reaction products micro/nano-particles present in fermented soybean sauce and vinegar

**DOI:** 10.1038/s41598-019-47800-6

**Published:** 2019-08-02

**Authors:** Suisui Jiang, Yanping Shi, Man Li, Liu Xiong, Qingjie Sun

**Affiliations:** College of Food Science and Engineering, Qingdao Agricultural University, Qingdao, Shandong Province 266109 China

**Keywords:** Nanoparticles, Chemical safety

## Abstract

The endogenous micro/nano-particles in daily food have drawn much attention due to specific properties potential biological impact. The aim of this study was to investigate the nanoparticles in traditional fermented soybean sauces and vinegars in order to study the safety problems of nanoparticles in daily food. The transmission electron microscope results showed that all samples exhibited diverse nanostructures with diameters ranging from 10 to 400 nm. The concentration of nanoparticles in these foods was determined to be around 1.15 × 10^7^–3.43 × 10^9^ particles/mL. Furthermore, the absorbance at 420 nm was found in all the fermented foods, which was ascribed to Maillard reaction products. The 3-(4, 5-Dimethylthiazol-2-yl)-2, 5-diphenyl-tetrazolium bromide (MTT) results showed that nanoparticles in traditional fermented foods did not decrease cell viability in the concentration range tested (<200 μg/mL), which were equivalent to 20 L~200 L of selected soybean sauces and vinegars. However, further studies need to be performed to find out the interaction of nanoparticle with cell (food with body) after ingestion.

## Introduction

In the current scenario, nanoparticles made from organic and inorganic materials have generated much interest, due to their unique physical and chemical properties and wide application potential^1–6^. In general, it is thought that inorganic nanoparticles are more toxic than biopolymer nanoparticles. Therefore, natural food components (e.g., chitosan, starch, lipid, and protein) have been widely employed to fabricate nanoparticles in recent years^7^. For example, the chitosan nanoparticle was investigated as a carrier for doxorubicin, and it was able to deliver doxorubicin into cells^8^. Lipid-based nanocarriers were fabricated to enhance solubility, improve bioavailability and ameliorate controlled release of phenolic compounds^9^. Resveratrol-loaded stearic acid-based solid lipid nanoparticles exhibited prolonged resveratrol release *in vitro* and an 8-fold improvement in oral bioavailability as compared to free resveratrol^10^. Caseinophosphopeptide-chitosan nanoparticles remained stable after incubation with digestion enzymes and exhibited significant anticancer activities^11^. Moreover, Nahar *et al*. reported that functional polymeric nanoparticles can be applied as an efficient and promising tool to carry active compounds, which would promote the increased efficacy of bioactive compounds^12^. Besides the engineered nanomaterials fabricated with food ingredients, natural nanoparticles present in foods or organisms have also been reported. For example, bovine milk contains widely dispersed colloidal particles composed mainly of casein micelles and colloidal calcium phosphate at an average of about 200 nm^13^. Lipid nanoparticles are present in soybean seed oil bodies, which is composed of protein-coated triglyceride droplets^14^. A nanostructure with a size range of 100–200 nm is found in cuttlefish ink melanin. Carbohydrate-based food caramels are found to consist of spherical nanoparticles^15^. These natural nanoparticles present in food items have the potential for application in the food and medical fields, and they possess similar attributes and present fewer risks to human health.

In China, vinegars and soy sauces as condiments are consumed in daily life, which are produced by fermentation of cereals. During thermal treatment and fermentation process, high amounts of Maillard reaction products (MRPs) including melanoidins are formed, which are contributed to flavor and color^16^. Melanoidins is formed mainly of carbohydrates, phenolic compounds, and protein, which has versatile functional properties and exhibits antioxidant, antimicrobial, and antihypertensive activities^17–20^. Inspired by the occurrence of nanoparticles present in cuttlefish ink melanin and food caramels, we hypothesize that melanoidins in fermented foods might exist in the form of nanoparticles. Therefore, we investigate the nanoparticle properties of traditional fermented foods containing melanoidin, such as sauce and vinegar. Of these foods, soy sauce and vinegar are traditional condiments consumed by the Chinese, Korean, and Japanese peoples. These foods are mainly prepared by the process of cereal fermentation, which leads to the formation of melanoidin. Therefore, the aim of this work is to study the safety problems of nanoparticles in traditionally fermented foods, which have been consumed for thousands of years.

## Results and Discussion

### Nanoparticle size distribution and concentration

**Figure 1:**
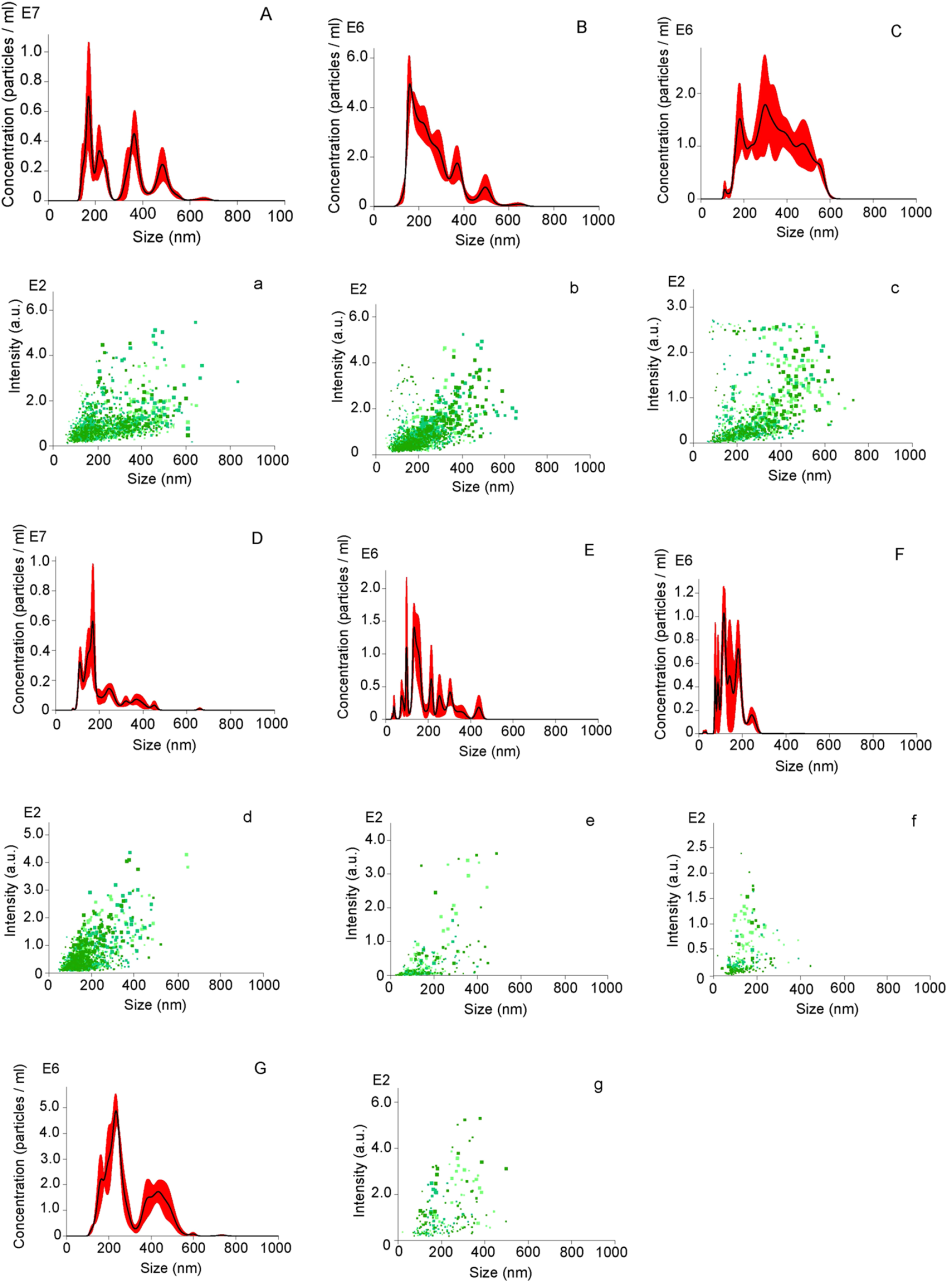
Concentration and size distribution of Luhua soybean sauce (**A**,a), vinegar (**B**,b), Haitian soy sauce (**C**,c), Zilin mature vinegar (**D**,d), Qiaoxifu sauce (**E**,e), vinegar (**F**,f), and melanoidin (**G**,g), respectively.

The size distribution and nanoparticle concentration of different brands of sauces or vinegars was calculated by nanoparticle tracking analysis (NTA), as shown in Fig. 1. The size of Luhua soybean sauce was in the range of 150–650 nm, whereas the concentration was determined to be around 7.95 × 10^8^ particles/mL (Fig. 1A). According to the statistical results of particle size dispersion analysis (Fig. 1a), the particle size of Luhua soybean sauce is mainly concentrated in the 100–400 nm range. Luhua vinegar’s particle distribution ranged widely from 80–700 nm, which was in accordance with a high PDI (Fig. 1B). Moreover, its concentration was 7.48 × 10^8^ particles/mL (Fig. 1b). A further investigation of the nanoparticle concentrations of other brands of sauces and vinegars was performed. NTA results showed that the concentrations of Haitian soy sauce and Zilin mature vinegars were 4.97 × 10^8^ and 4.78 × 10^8^ particles/mL, respectively (Table 1). Compared to the other brand sauces, the size distribution range of Qiaoxifu sauce was wider, indicating that the nanoparticle distribution range was unequal (Fig. 1E,e). This result was in agreement with the polydispersity index (PDI). Additionally, the concentration of the Qiaoxifu sauce and vinegar were significantly lower in comparison with other fermented foods (Fig. 1e,f) (Table 1). The MRPs prepared by glucose and aspartic acid were also evaluated. The results showed that the size of the MRPs were mainly concentrated in the range of 100–400 nm (Fig. 1g), and its concentration was 7.48 × 10^8^ particles/mL. The nanoparticle concentration of the MRPs was far less than that of the other natural fermented foods.

**Table 1:** Parameters of natural nanoparticles present in different brands of sauces and vinegars.

Sample	Mean diameter (nm)	PDI	Concentration ( × 10^8^ particles/mL)
Luhua soybean sauce	306.1 ± 7.5	0.46 ± 0.05	7.95 ± 0.67
Luhua vinegar	263.2 ± 18.0	0.54 ± 0.19	7.48 ± 0.56
Haitian soy sauce	344.0 ± 19.3	0.53 ± 0.02	4.94 ± 0.05
Zilin mature vinegar	207.2 ± 11.9	0.11 ± 0.02	4.78 ± 0.23
Qiaoxifu sauce	396.6 ± 1.6	0.85 ± 0.11	1.19 ± 0.23
Qiaoxifu vinegar	148.1 ± 5.5	0.38 ± 0.04	0.74 ± 0.01
melanoid	236.0 ± 28.9	0.46 ± 0.01	0.12 ± 0.02

**Figure 2:**
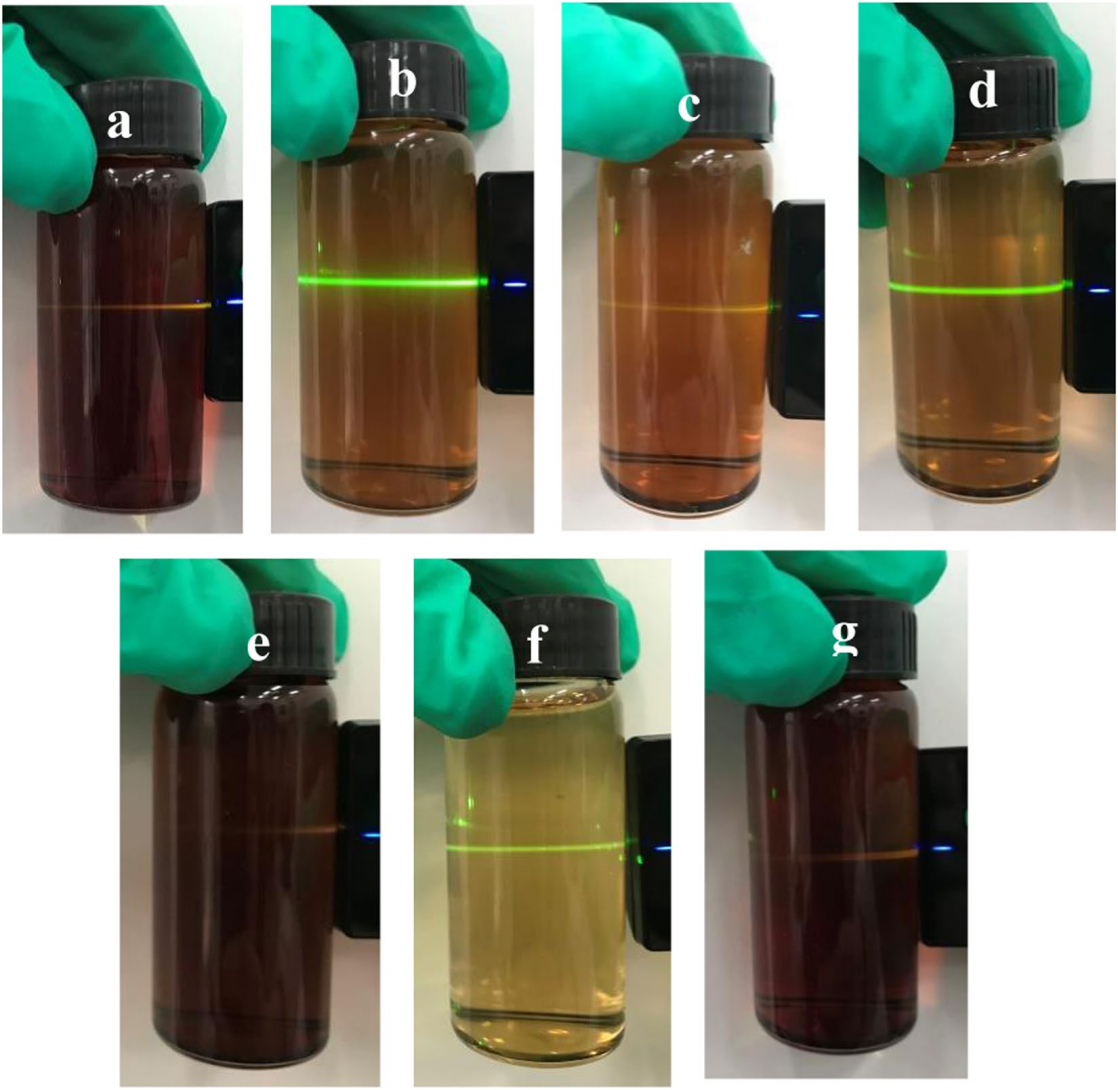
Tyndall effect of Luhua soybean sauce (**a**), vinegar (**b**), Haitian soy sauce (**c**), Zilin mature vinegar (**d**), Qiaoxifu sauce (**e**), vinegar (**f**), and melanoidin (**g**) dispersions in water, respectively. Sauces and vinegars were diluted 10 times with Milli-Q water.

To further investigate whether there were actual nanoparticle dispersions of these natural fermented foods in water, the Tyndall effect test was employed to identify their properties. A manifest laser beam going through the dispersion could be observed while the laser beam irradiated the dispersion, as shown in Fig. 2. In other words, the Tyndall effect took place in the MRPs, different brands of sauces and vinegars. Nevertheless, Qiaoxifu sauce did not exhibit the Tyndall effect, which was presumably due to the heterogeneous particles in this system. This result coincided with a high PDI and large mean diameter. Furthermore, a uniform dispersion of the MRPs, different brands of sauces and vinegars could be maintained for a certain period of time. The result of Tyndall effect test suggested that MRPs and natural fermented foods could form the stable dispersion which has been ascribed to nanoparticles in these systems.

### Characterization of natural fermentation food nanoparticles

**Figure 3:**
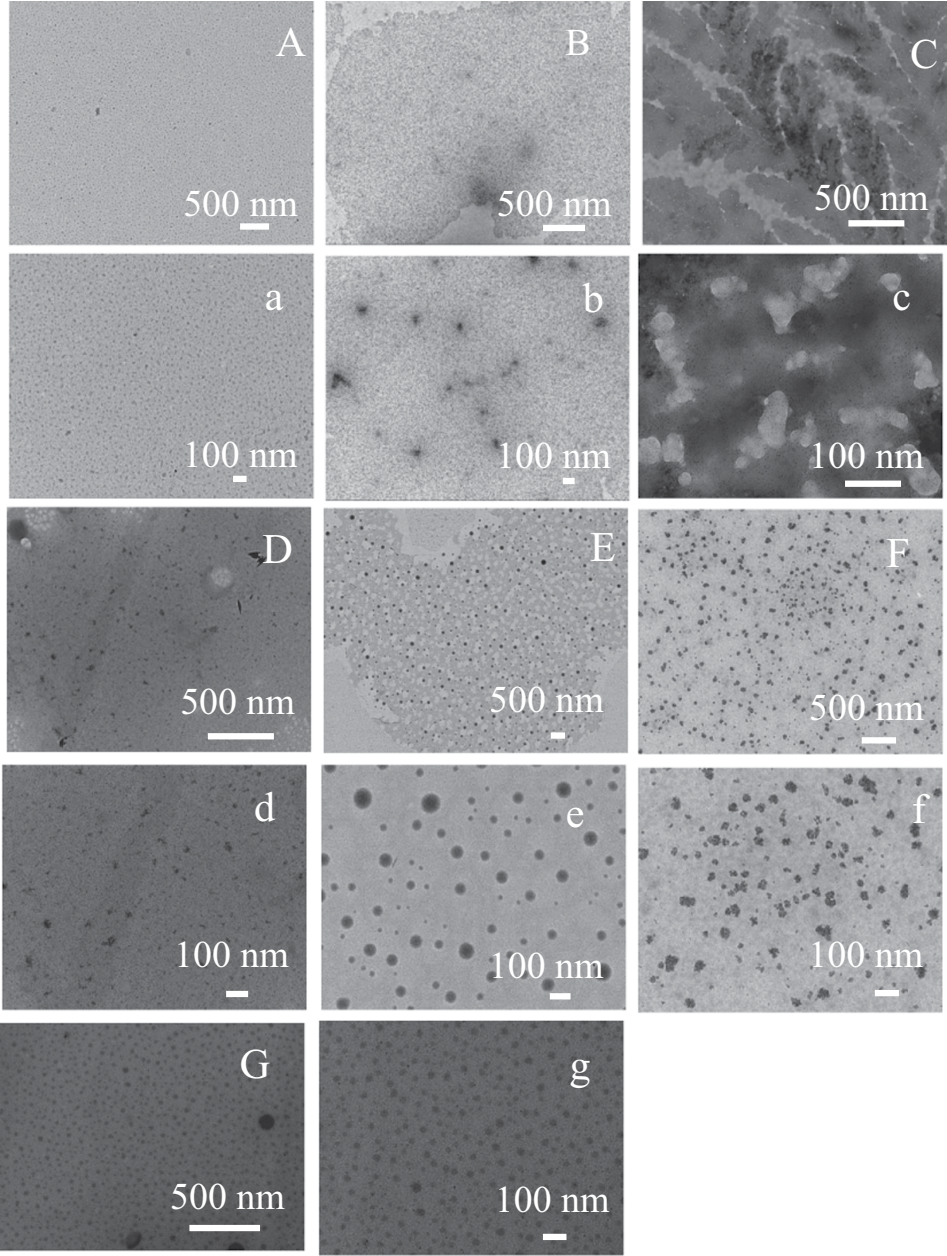
TEM images of Luhua soybean sauce (**A**,a) and vinegar (**B**,b), Haitian soy sauce (**C**,c), Zilin mature vinegar (**D**,d), Qiaoxifu sauce (**E**,e), vinegar (**F**,f), and melanoidin (**G**,g) nanoparticles with lower (capital letter) and higher magnification (lowercase letter).

TEM was used to determine the morphology and size of nanoparticles from different brands of sauces or vinegars; the results are presented in Fig. 2. Different brands of sauces or vinegars exhibited diverse nanostructures. The Luhua soybean sauce exhibited spherical morphology with a diameter of about 30 nm (Fig. 3A,a). In particular, single nanoparticles from Luhua soybean sauce dispersed uniformly. Nanoparticles of Luhua vinegar were heterogeneous with a wide size distribution (Fig. 3B,b). A further investigation on whether the other brands of sauces and vinegars contained nanoparticles was conducted. Haitian sauce, Zilin mature vinegar, and Qiaoxifu sauces or vinegars were employed. As shown in Fig. 3C, window patterns in winter (ice-flower) were found in Haitian soy sauce, and no obvious nanostructures were observed. However, small nanoparticles and agglomerates could be observed at the higher magnification (Fig. 3c). The morphologies of Zilin mature vinegar were of irregular nanostructure, as depicted in Fig. 3D,d. In contrast, the Qiaoxifu sauce nanoparticles were heterogeneous with diameters in the range of 30–100 nm (Fig. 3E,e). It could be clearly seen that the nanoparticles were regular spheres compared to those of the abovementioned samples. Additionally, corresponding flower-like structures were found in Qiaoxifu vinegar (Fig. 3F,f). The nanoparticles existing in these natural foods were probably due to MRPs formed during the fermentation process. To prove this hypothesis, MRPs were fabricated and then dialyzed to remove impurities. Spherical nanoparticles were found with a diameter of around 30 nm (Fig. 3G,g). Similarly, Palashuddin Sk *et al*. found that carbon dots in the range of 4–30 nm were formed in different carbohydrate-based food caramels during the heating process^15^. The size of nanoparticles is larger than that of carbon dots derived from roasted Chicken^21^.

The particle size distributions and PDI of nanoparticles in the fermented foods were monitored by dynamic light scattering (DLS), and the results are shown in Table 1. The average size and PDI varied for different brands of sauces or vinegars. For all samples, the average size was 100–300 nm, except for Qiaoxifu sauce. Meanwhile, all samples had a relatively high PDI, indicating the broad distribution of these natural fermented foods. The reason for this may be that the fermentation conditions and technologies of the manufacturers were different or that the variation of the main constituents in the grain was heterogeneous during the fermentation process.

### UV-Vis spectral features of fermented foods

**Figure 4:**
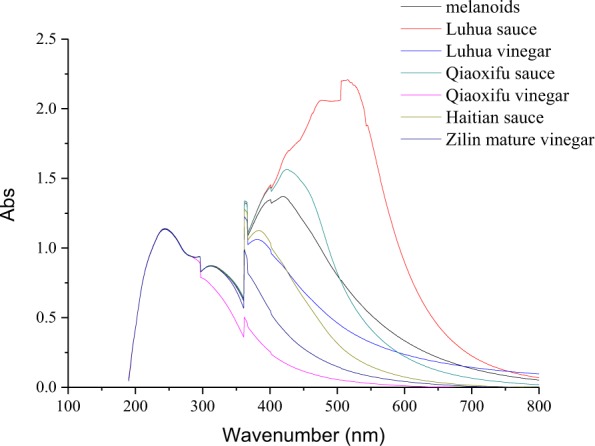
UV-Vis absorption spectra of Maillard reaction products and different brands of sauces and vinegars nanoparticles.

The UV-Vis spectra of natural fermented foods were monitored by UV-Vis absorption, and the results are shown in Fig. 4. The spectra of samples from fermented soybean foods (sauces and vinegars) showed a characteristic absorption peak at around 330 nm, which was derived from the intermediate MRPs formed during the fermentation process. There was also a characteristic peak at 440 nm, which was derived from the final stage products of the melanoidin during the fermentation process. Moreover, the melanoidin compounds prepared from aspartic acid and glucose through Maillard reaction also showed a similar absorption at 420 nm, which has been widely reported by several studies^22,23^. The results indicated that the main component of the nanoparticles in these samples might be the Maillard reaction products. Also, melanoidins from sauce and vinegar exhibit a high antioxidant activity^18,24–27^. In other words, melanoidin nanoparticles from sauce and vinegar are not only safe, but also bioactive, and they can be used as a safe and promising material.

### Evaluation of cell viability

**Figure 5:**
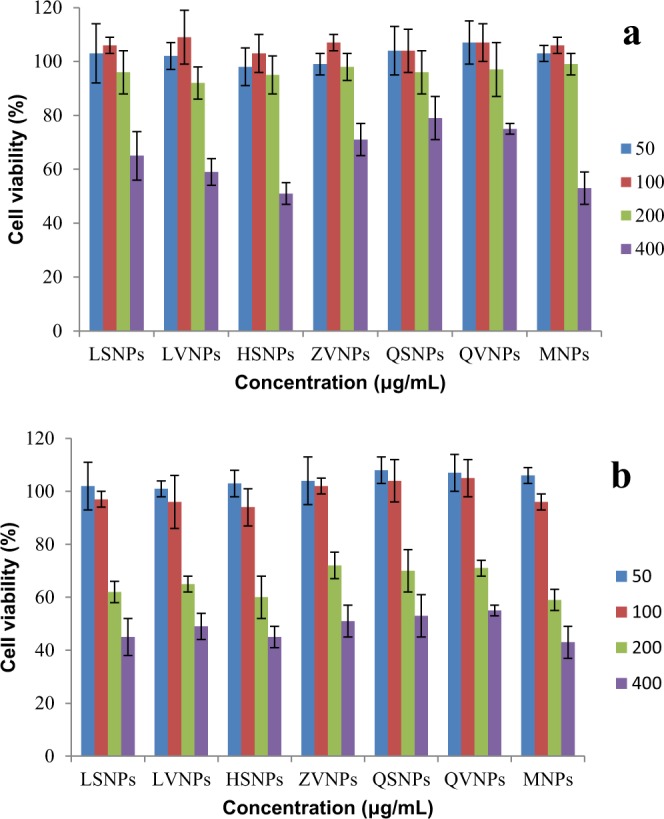
Influence of Luhua soybean sauce (LSNPs), Haitian soy sauce (HSNPs), Qiaoxifu sauce (QSNPs), Luhua vinegar (LVNPs), Zilin mature vinegar (ZVNPs), Qiaoxifu vinegar (QVNPs), and melanoidin (MNPs) nanoparticles on the viability of human intestinal epithelial cells Caco-2 cells. Incubation time was 12 (**a**) and 24 h (**b**), and nanoparticles concentration ranged between 50 and 400 μg/mL.

In order to study the safety of nanoparticles in fermented food, it is essential to ensure whether the nanoparticles from fermented food have cytotoxic to cells. Therefore, the cytotoxicity of nanoparticles on Caco-2 cells was assessed by MTT assy. As observed in Fig. 5, the nanoparticles showed a dose-dependent manner on the tested cells. Neither various brand sauces and vinegars nanoparticles show any toxicity to Caco-2 cells at concentration blow 200 μg/mL for 12 h, which were equivalent to 20 L~200 L of selected soybean sauces and vinegars. Decease in cell viability was observed when the cells were incubated with nanoparticles at 400 μg/mL for 12 h. The differences between the cytotoxicity of different nanoparticles may be ascribed to differences in the size and charge density^28,29^. As shown in Fig. 5b, nanoparticles reduced the cell activity at 200 μg/mL for 24 h. The cell viability was ~95% at 200 μg/mL for 12 h and only 65% at 200 μg/mL for 24 h. Similarly, MgO nanoparticles resulted in different cell activity at same concentration for different incubation time^30^. The reason why the different results were observed might be because the differences of nanoparticles accumulation in cells at different time. Thus, to avoid the toxic effect induced by nanoparticles from fermented food, the concentration of nanoparticles used in food should less than 200 μg/mL that

### Mechanism illustration of formation of nanoparticles during the fermentation process

**Figure 6:**
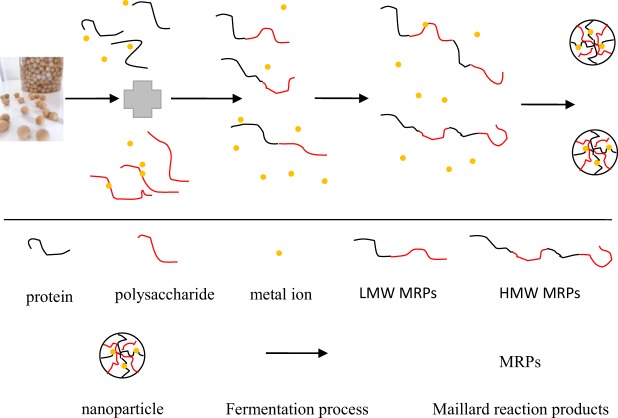
Schematic illustration of the formation of nanoparticles in the food fermentation process. Low molecular weight (LMW); high molecular weight (HMW).

During the fermentation and production processes, such as the heat treatment of raw materials and pasteurization of products, carbohydrate abundance in grain could react with protein to form Maillard reaction products, including melanoidins, which is the main component in the brown color and flavor. During thermal treatment, a large number of colorless aroma compounds, ultra-violet absorbing intermediates, and dark-brown polymeric compounds are produced. Additionally, in the early fermentation stage, the amino acid and glucose content increased with increasing fermentation time, and a few Maillard reaction products with low molecular weight (LMW) were formed (Fig. 6). Then, the Maillard reaction further occurred and a large amount of Maillard reaction products with high molecular weight (HMW) were produced by cross-linking chromophoric LMW Maillard reaction products and reactive amino acid side chains. At this stage, the system became stable. Maillard reaction products, especially melanoidins, are capable of binding to metal ions^31,32^, and have a potential tendency to assemble into nanodots. Subsequently, an alternative aggregation pathway was followed which resulted in nanoparticles.

## Conclusions

In summary, we first discovered the occurrence of natural nanoparticles in commonly consumed soybean sauces and vinegars. Different fermented foods exhibited diverse nanostructures, including spherical morphology, irregular nanostructure, and flower-like structure. The concentration of nanoparticles in different brands of soybean sauces or vinegars was determined to be around 1.15 × – 3.43 × 10^9^ particles/mL. The nanoparticles were probably due to MRPs which self-assembled in the fermentation process. In addition, the nanoparticles were found to be non-cytotoxic up to a concentration of 200 μg/mL, which were equivalent to 20 L~200 L of selected soybean sauces and vinegars. Both of soybean sauces and vinegar are condiments that people eat few in daily life, so there is no risk to human health during daily consumption. Therefore, the natural nanoparticles reported in our work are a safe and promising nanomaterial under experimental condition, which has potential applications in the food and medical fields. However, more research on the isolation of Maillard reaction products nanoparticles and the construction of nanocarrier systems for active substances will be required for their effective use in various fields.

## Materials and Methods

### Reagents and materials

Luhua soybean sauce, Haitian soy sauce, Qiaoxifu sauce, Luhua vinegar, Zilin mature vinegar, and Qiaoxifu vinegar were purchased from the RT-MART market (Qingdao, China). Aspartic acid and glucose were supplied by the Sinopharm Group Co., Ltd. (Shanghai, China). 3-(4, 5-Dimethylthiazol-2-yl)-2, 5-diphenyl-tetrazolium bromide (MTT) was purchased from Gibco-BRL (Bethesda, MD, USA).

### Purification of nanoparticles

To obtain the nanoparticles content in initial samples, various brand sauces and vinegars (10 mL) were diluted with 90 mL ethanol (100%) at room temperature for 12 h. After removing the solid precipitate, the ethanol volume was reduced to 1 mL in a rotary evaporator. Then obtained product was dissolved in 100 mL distilled water and dialyzed using dialysis membrane with a nominal 1000 Da cutoff (YA1065, Solarbio life sciences Corp., Beijing, China) against water for 48 h to remove the water soluble impurities.

Maillard reaction products were prepared with some modifications according to a previously reported procedure^22^. Aspartic acid (1 mg/mL) was mixed with a glucose (1 mg/mL) solution in glass tubes, and then heated at 120 °C for 2 h. The reaction mixture solution was then transferred to a dialysis bag with a nominal 1000 Da cutoff. The dialysis bag was immersed in Milli-Q water for 48 h to remove impurities and freeze-dried (Scientz-10ND, Ningbo Scientz Biotechnology Co. Ltd., Ningbo, Zhejiang, China).

### Nanoparticle tracking analysis

Various brand sauces and vinegars were diluted with Milli-Q water (sample: water = 1:9, v/v), and then dialyzed against water for 24 h. The nanoparticle size distribution and concentration in the samples were determined using the nanoparticle tracking analysis (NTA) method on the Malvern NanoSight NS300 (Malvern Paralytics, Malvern, UK) with a flow-cell top plate and a 405 nm laser. Automatic settings for the maximum jump distance and blur settings were utilized. The detection threshold for all samples was 5. Data were analyzed on the NTA software 3.0.

### Tyndall effect

The Tyndall effect was investigated using a laser pointer (2804; Deli Group Co., Ltd., Ningbo, China) with a wavelength of 532 nm. A laser beam was focused into the dispersions of each sample.

### Transmission electron microscope (TEM)

The morphology of the nanoparticles in fermented food samples (sauces, vinegars, and melanoidin) was recorded using a 7700 TEM (Hitachi, Tokyo, Japan) operating at an acceleration voltage of 80 kV. A tiny drop of nanoparticle dispersion (20 μL) was deposited on a carbon-coated copper grid, and excess water was blotted with filter paper. Freeze-dried samples were observed.

### Size distribution and polydispersity index (PDI)

The particle size distribution and PDI of the nanoparticles were measured via dynamic light scattering (DLS) (Zetasizer Nano ZS90; Malvern Panalytical, Malvern, UK). The intensity of light scattering was monitored at a 90° angle, and the sample suspensions were analyzed at 25 °C.

### Ultraviolet-vis (UV-Vis) absorption measurements

The UV-Vis analysis of the nanoparticles was performed using a UV-vis spectrophotometer (TU-1810; Beijing Persee Co., Ltd., Beijing, China) in the continuous range of 200–800 nm using a quartz cuvette (1 cm path length) at 1 nm intervals. The final spectra were baseline corrected by subtracting the deionized water spectra.

### MTT cytotoxicity assy

Cytotoxic activities of nanoparticles from fermented food were determined by MTT assay according to Karamanidou *et al*.^33^. Caco-2 and Raw264.7 were grown and maintained in DMEM medium supplemented with 10% FBS, 100 U/mL of at penicillin and 100 μg/mL of streptomycin at 37 C in an atmosphere of 5% CO_2_. After reaching 80% confluence, the cells were plated at a cell density of 5 × 10^4^ cell/well into 96 well plates. After 24 h incubation, the medium was removed and 200 μL nanoparticles were added. The final concentration of nanoparticles varied in the range of 0, 25, 50, 100, 200, 400 μg/mL. After incubation for 2 and 12 h, the cells were washed twice by PBS. Then 50 μL MTT solution (2 mg/mL) was added to each well. After further incubation for 4 h, 150 μL DMSO was added to each well to dissolve the formazan crystals. The absorbance was measured at 570 nm. The cell viability was calculated according to the following equation:

$${\rm{Cell}}\,{\rm{viability}}\,( \% )={{\rm{Abs}}}_{{\rm{sample}}}/{{\rm{Abs}}}_{{\rm{control}}}\times \mathrm{100} \% $$

In all assays, the unexposed cells were evaluated as the negative control.

### Statistical analysis

The treatments were done in triplicate, with values reported as the means ± SD. All the differences were considered to be significant at *P* < 0.05.

## Data Availability

This article is licensed under a Creative Commons Attribution 4.0 International License, which permits use, sharing, adaptation, distribution and reproduction in any medium or format, as long as you give appropriate credit to the original author(s) and the source, provide a link to the Creative Commons license, and indicate if changes were made.
